# Burrowing hard corals occurring on the sea floor since 80 million years ago

**DOI:** 10.1038/srep24355

**Published:** 2016-04-14

**Authors:** Asuka Sentoku, Yuki Tokuda, Yoichi Ezaki

**Affiliations:** 1 Seto Marine Biological Laboratory, Field Science Education and Research Center, Kyoto University, 459 Shirahama, Nishimuro, Wakayama 649-2211, Japan; 2 Tottori Prefectural Museum, 2-124 Higashimachi, Tottori 680-0011, Japan; 3 Department of Geosciences, Faculty of Science, Osaka City University, 3-3-138 Sugimoto, Sumiyoshi-ku, Osaka 558-8585, Japan; 4 Tottori University of Environmental Studies, 1-1-1 Wakabadaikita, Tottori 689-1111, Japan

## Abstract

We describe a previously unknown niche for hard corals in the small, bowl-shaped, solitary scleractinian, *Deltocyathoides orientalis* (Family Turbinoliidae), on soft-bottom substrates. Observational experiments were used to clarify how the sea floor niche is exploited by turbinoliids. *Deltocyathoides orientalis* is adapted to an infaunal mode of life and exhibits behaviours associated with automobility that include burrowing into sediments, vertical movement through sediments to escape burial, and recovery of an upright position after being overturned. These behaviours were achieved through repeated expansion and contraction of their peripheral soft tissues, which constitute a unique muscle-membrane system. Histological analysis showed that these muscle arrangements were associated with deeply incised inter-costal spaces characteristic of turbinoliid corals. The oldest known turbinoliid, *Bothrophoria ornata*, which occurred in the Cretaceous (Campanian), also possessed a small, conical skeleton with highly developed costae. An infaunal mode of life became available to turbinoliids due to the acquisition of automobility through the muscle-membrane system at least 80 million years ago. The newly discovered active burrowing strategies described herein provide new insights into the use of an unattached mode of life by corals inhabiting soft-bottom substrates throughout the Phanerozoic.

Due to their sessile lifestyles, scleractinian corals are vulnerable to being covered by deposited sediments^1^. Since being covered by sediments is typically fatal for corals, sediment deposition is a major factor affecting coral distribution. Despite this vulnerability to sedimentation, 330 species of azooxanthellate scleractinian corals, or approximately 22% of all scleractinians, live on deep-water sandy and/or muddy substrates where the risk of burial and smothering is high^2^. Given the difficulties associated with accessing, sampling, and observing these coral species, little is known about the ecological traits and adaptation mechanisms employed by these corals for living in soft substrate environments.

The family Turbinoliidae (Cnidaria: Scleractinia) is composed exclusively of free-living, solitary, azooxanthellate corals that inhabit soft-bottom substrates for at least their full grown or anthocyathus stage after detachment^3^. Turbinoliids typically measure less than 1 cm in calicular diameter and have conical, bowl-shaped, or cylindrical forms. The family exhibits high levels of diversity, with 23 Recent and 6 fossil genera identified from the Late Cretaceous (Campanian) approximately 80 Ma onwards^3^,^4^,^5^. Despite this diversity, however, no formal investigations have been conducted on living turbinoliids and little is known about their modes of life and life history traits^3^. The conical morphology was used to infer that it had a semi-burrowing mode of life analogous to that observed in sand-burrowing sea anemones (Actiniaria)^6^. Additionally, with its very small corallum, *Sphenotrochus* was inferred to be an interstitial sedentary (not automobile) dweller in moderately shallow and reworked coarse sandy substrates^4^,^7^,^8^. However, even if most, if not all, turbinoliids are semi-burrowers or interstitial dwellers, in the absence of direct observations of the behaviours of living specimens, any interpretations of their modes of life remain speculative.

The present study therefore examined the burrowing, escaping, and righting (turning over) behaviours of the turbinoliid *Deltocyathoides orientalis* Duncan, 1876 based on observations of living individuals. This is the first study to present evidence of active burrowing and infaunal modes of life in the Scleractinia.

## Results

**Figure 1:**
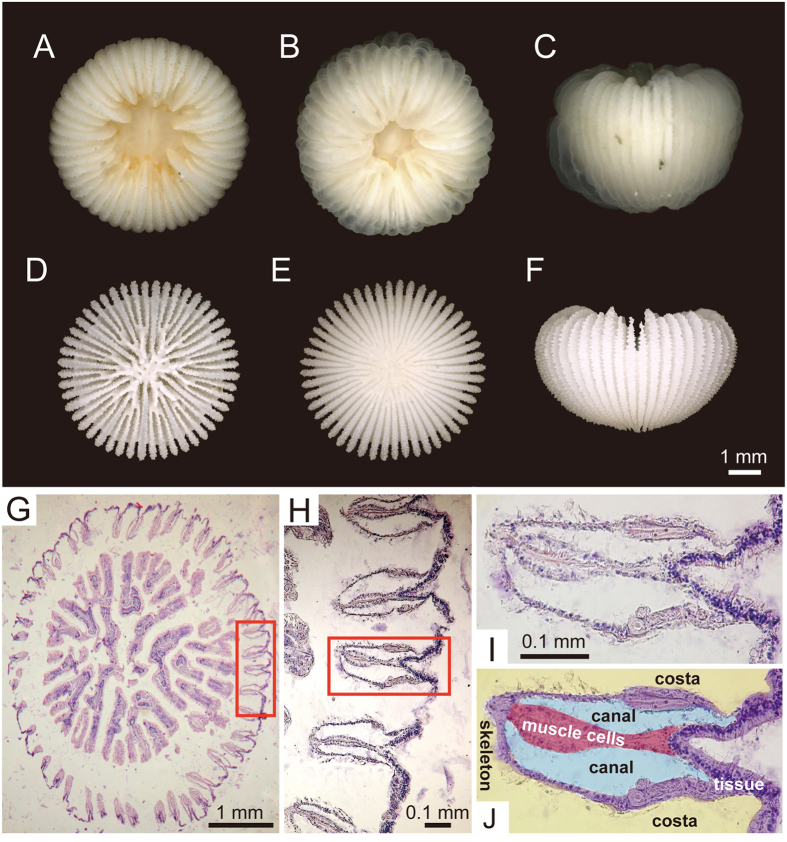
Morphological features of *Deltocyathoides orientalis*. (**A**–**C**) living soft parts. (**A**) top view. (**B**) top view of swollen soft parts. (**C**) lateral view. (**D**–**F**) corallum skeleton. (**D**) top view. (**E**) bottom view. (**F**) lateral view. (**G**–**J**) transverse slices of *D. orientalis*. (**H**) enlargement of the red rectangle indicated in (**G**), indicating the epidermal and gastric layers. (**I**–**J**) enlargement of the red rectangle in (**H**).

*Deltocyathoides orientalis* inhabits soft-bottom substrates and possesses a hemispheric or bowl-shaped skeleton completely covered by soft tissue (Fig. 1A–F). The mouth is located in the centre of the upper part of the corallum (calice) and is encircled by 48 tentacles, which are swelled due to the intake of ambient water. The soft parts covering the exterior of the curved skeleton are composed of a two-fold membrane that is partitioned by sheet-like muscles, and the divided inter-membranous parts (=canals) are filled with the water (Fig. 1G–J; Supplementary Fig. S1). Rapid expansions of the polyp at the oral or aboral sides are precisely controlled by abrupt changes in fluid pressure within the canals, which is generated by contraction of opposite side of the polyp (Supplementary Fig. S1C–E).

**Figure 2:**
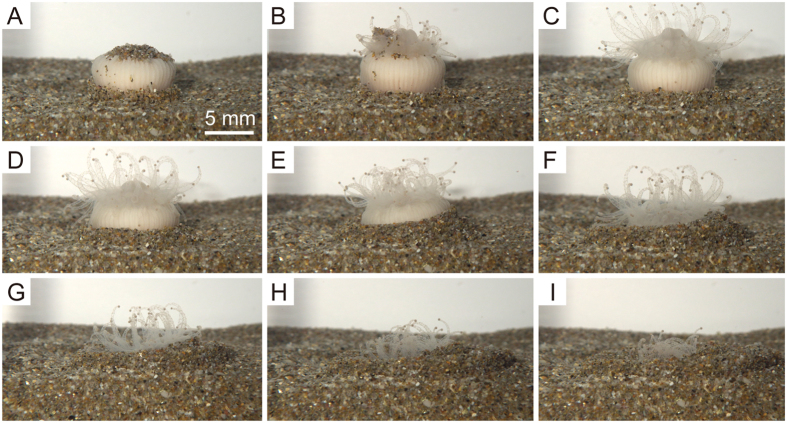
Time-lapse series of a burrowing polyp. (**A**) initial placement of the coral on a sand substrate (0 min). (**B**) elapsed time 30 min. (**C**) elapsed time 90 min. (**D**) elapsed time 210 min. (**E**) elapsed time 330 min. (**F**) elapsed time 450 min. (**G**) elapsed time 570 min. (**H**) elapsed time 690 min. (**I**) elapsed time 810 min.

**Figure 3:**
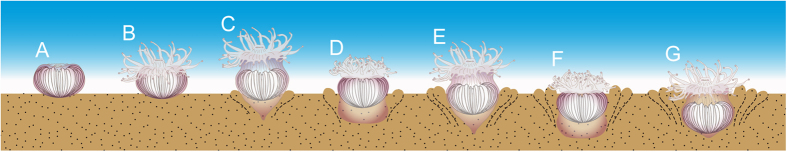
Schematic diagram depicting a time-lapse series of a polyp burrowing into a soft-bottom substrate and exhibiting soft-part behaviours characteristic of *Deltocyathoides orientalis*. (**A**) initial placement of a coral on a sand substrate. (**B**) expansion of tentacles. (**C**–**F**) gradual expansion of the polyp’s aboral base results in the upward and outward lateral shedding of adjacent sediments. (**C**,**E**) the polyp’s aboral base assumes the shape of a horn so as to dig itself into the substrate and shed adjacent sediments laterally. (**D**,**F**) seawater stored in the interior of the soft parts moves to the aboral side of the polyp through canal over the skeleton powered by contraction of the polyp. (**G**) the corallum is buried with its tentacles extended above the substrate.

**Figure 4:**
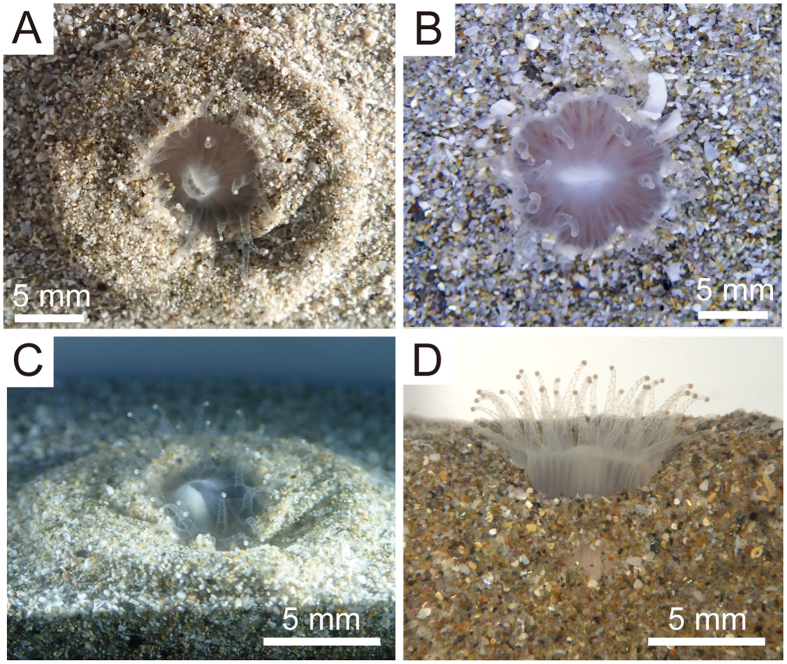
Life position on soft-bottom substrates and disc-like burrowing depressions of *Deltocyathoides orientalis*. (**A**,**B**) top views during burrowing. (**C**,**D**) oblique and lateral views during burrowing. Note that coralla are buried with their tentacles extended above the substrate.

Burrowing behaviour in *D. orientalis* was observed to occur as follows (Fig. 2; Supplementary Movies S1 and S2): (1) The aboral part of the polyp assumed to have a conical shape as the part was extended downward and pushed some of the adjacent sediment laterally (Figs 2A,B and 3A–C). (2) The oral part of polyp then swelled due to the absorption of seawater into the body (Figs 2C and 3C), and seawater stored in the interior of the polyp moved towards the aboral base through the inter-membranous canals via contraction of the oral side of polyp followed by rapid expansion of the aboral part (pumping system; Fig. 3D). This expansion of the aboral part, in turn, caused the lateral displacement of sediments in a concentric pattern around the polyp. (3) Following expansion of the aboral part and subsequent transport of seawater through the inter-costal canals back up towards the oral part of the polyp, the aboral base returned to its normal size (Fig. 3E). In this way, the burrowing movement proceeded step by step, allowing the aboral base to gradually occupy the vacant space formed by the upward movement of substrate (Fig. 3F; Supplementary Fig. S2). This behaviour resulted in substrate sediment being transported upwards along a diagonal path as well as away from the polyp laterally (Supplementary Fig. S2). This repeated contraction and expansion of the polyp occurred at approximately 30-min intervals, which translated to a burrowing speed of approximately 0.8 mm/h. Immediately after complete burial of its corallum, it subtly repositioned itself within the substrate (Fig. 3G). Once the whole corallum was buried completely, the polyp extended its tentacles to acquire food just above the substrate (Fig. 4). Upon physical stimulation of the extended tentacles, the polyp retracted abruptly into the substrate, withdrawing the soft parts into the calice.

When overturned, the aboral part of the *D. orientalis* coral assumed a conical shape before being inflated by the intake and transport of seawater through the inter-costal canals (Fig. 3A–C). Seawater retained in the aboral part of polyp was then transported via contraction of the soft parts towards the oral end of the polyp (Supplementary Fig. S3D). Repeated expansion of oral part of polyp tilted the corallum obliquely (Supplementary Fig. S3E,F), causing a rotation of the corallum in the horizontal axis (Supplementary Fig. S2G; Supplementary Movie S3), allowing the corallum to gradually return to its upright position, and resumed burrowing into the substrate (Supplementary Fig. S3H,I).

In order to observe the response of *D. orientalis* to being buried completely, a corallum was covered with approximately 10–15 mm of additional sandy sediment (three to four times the corallum height) (Supplementary Fig. S4A; Supplementary Movie S4). Approximately 90 min after the complete burial of the corallum, a series of repeated upward and downward movements of the sediment were detected at the substrate surface immediately above the corallum. These sediment movements were attributed to the repeated expansion and subsequent contraction of the oral and aboral ends of the polyp at intervals ranging from 20–40 min, respectively (Supplementary Fig. S4B). Repeated expansion of the oral end forced the polyp upwards into the sediment above the corallum (i.e. towards the surface). Subsequent expansion and wedging of the elongated oral end of the polyp followed by contraction of the retractor muscles resulted in the entire corallum being pulled upward through the substrate (Supplementary Fig. S4C). Approximately four hours after being buried, the tips of the tentacles began to protrude from the surface of the substrate (Supplementary Fig. S4D), followed by the appearance of the entire oral side of polyp (Supplementary Fig. S4E,F). The vertical movement of the coral through the sediment resulted in the formation of a funnel-shaped sedimentary structure (Supplementary Fig. S4C–F). The mean speed at which coralla escaped being buried was 1.74 ± 0.2 mm/h (mean ± S.E.; n = 6).

## Discussion

The behaviours of lateral migration, escape from burial, and righting action have been reported previously in free-living fungiid corals using time-lapse imaging techniques^9^,^10^,^11^. However, burrowing behaviour has not yet been observed in hard corals. The acquisition of automobility in *D. orientalis*, including burrowing, escape from burial, and righting behaviours, implies that the species is actively utilising habitat under the sea-floor. The ability of *D. orientalis* to retract their oral side of the polyp into sediments is considered to be an anti-predator response like burrowing sea anemones and tube dwelling anemones^12^,^13^,^14^,^15^. The small size and light weight of *D. orientalis* coralla facilitate automobility via hydrostatic pressure applied through expansion of the polyps. Incised inter-costal areas allow canals to transport water along the vertical axis through alternate contraction and expansion of the polyps. The longitudinally oriented sheets of muscle cells in these inter-costal areas are considered to play an important role in regulating this expansion and contraction during burrowing, escaping from burial, and righting behaviours.

The turbinoliids are all free-living corals that are completely covered by soft tissue around the skeleton for at least the full-grown or anthocyathus stage after detachment. The family Turbinoliidae is comprised of cylindrical (3 genera), bowl-shaped (2 genera), or conical forms (23 genera). Turbinoliids are typically smaller than 10 mm in calicular diameter and have highly developed costae^3^,^4^,^5^. Prior to the present study, the functional significance of these costae was unknown. Histological examination of *D. orientalis* demonstrated that the sheet-like muscles covering the corallites controlled the expansion and contraction of the outer polyp during burrowing, escaping from burial, and righting behaviours. The costae of turbinoliids protect these muscle sheets during contraction and facilitate their contractile force by increasing their surface area. The oldest known turbinoliid, *Bothrophoria ornata* Felix, 1909, which occurred in the Cretaceous (Campanian), also possessed a small, conical skeleton with highly developed costae^3^,^4^. This structural specialization, which would have enabled this species to burrow into soft-bottom substrates, may have facilitated the high diversity of the Turbinoliidae. Although this infaunal mode of life in soft-bottom substrates was previously unrecognized, the presence of these traits in the fossilized remains of *B. ornata* implies that the infaunal mode has been utilised by the Scleractinia for at least 80 Ma. These findings also suggest that costae and small coralla are traits among turbinoliids essential for their burrowing behaviours.

Small coralla with highly developed costae as seen in turbinoliids are also found in the extinct orders Rugosa and Tabulata, which existed during the Palaeozoic. A few groups within these extinct orders are also considered to have exhibited automobility^16^,^17^. For example, the Devonian rugose coral *Hadrophyllum asturicum* was morphologically very similar to *D. orientalis*^18^, implying that *H. asturicum* may have occupied a similar ecological niche.

The discovery of burrowing scleractinians is very intriguing since it shows that adaptive radiation of hard corals occurred through the positive utilisation of space under the sea floor, in spite of their vulnerability to sediment. Most importantly, information regarding the exploitation of infaunal niches provides invaluable insights into the life history of marine benthic animals throughout the Phanerozoic.

## Methods

We examined 15 individuals of *Deltocyathoides orientalis* (Fig. 1), which were collected at depths of 94–115 m off the Pacific coasts of Ohakozaki (39°21.96611 N, 142°01.5922 E) and Hachinohe (40°30.0862 N, 141°50.8544 E), Japan. Of these, 6 living individuals were selected for observation and morphometric measurements. Greater calicular diameter and corallum height ranged from 3.6 to 8.4 mm and 1.6 to 4.9 mm, respectively.

Individuals selected for analysis were maintained for more than 10 months in an acrylic tank (65 cm long, 28 cm wide and 35 cm high) fitted with a mechanical particle filter and a thermostat-controlled cooling system (ZC-1300E; Zensui, Japan). The tank was filled with salty groundwater pumped up from a depth of approximately 50 m at a site located on the grounds of the Tottori Prefectural Farming Fisheries Center. Water temperature within the tank was maintained at 12 °C, which corresponded to temperatures observed in the natural habitat of the coral. Corals were cultured in the dark and fed frozen copepods once a week. The bottom of the tank on which the corals were placed was covered with either sand or fine silica substrates. All experimental trials were conducted in triplicate in small rectangular experimental chamber (11 cm high, 11 cm long and 3.5 cm wide) located within the plastic tank described above. Burrowing, escaping from burial, and righting behaviours were recorded by time-lapse photography using a digital camera (RICOH-WG-30) set in front of an acrylic board. Behavioural analysis of photographs was carried out using time-lapse movies viewed with Panolapse (v. 1.20) software.

The first experiment examined burrowing behaviour, and involved placing corals with their oral discs oriented upward on a plane surface comprised of a fine sand substrate. The second experiment examined the movement of sand particles around burrowing corals, and involved the placement of corals on a surface comprised of layered substrates consisting of white-, yellow-, blue-, and green-coloured medium- to fine-grained sand particles. Separate layers of sand were poured into the experimental chamber to produce less than 5 mm-thick planar laminae with sharp horizontal boundaries between them. The third experiment examined the reaction of corals to being buried in sediment. Corals that had already burrowed into the substrate were completely covered with the same coloured sand. Additional layers of differently coloured sand were then added to give a total thickness of 10 to 15 mm of substrate above the buried polyp (three to four times the average height of individual corals).

For histological analysis, *D. orientalis* polyps were pre-fixed with 99% ethanol followed by fixation and decalcification in Bouin’s solution for 24 h. Decalcified tissues were then rinsed in 70% alcohol, dehydrated in a graded alcohol series, and embedded in paraffin. Histological sections 5 to 6 μm in thickness were cut perpendicular to the oral-aboral axis with a microtome (Yamato Kohki Industrial Co., Ltd.) and then stained with haematoxylin and eosin.

## Additional Information

**How to cite this article**: Sentoku, A. *et al.* Burrowing hard corals occurring on the sea floor since 80 million years ago. *Sci. Rep.* **6**, 24355; doi: 10.1038/srep24355 (2016).

## Supplementary Material

Supplementary Information

Supplementary Movie S1

Supplementary Movie S2

Supplementary Movie S3

Supplementary Movie S4
