# Supplementary material for: Burrowing hard corals occurring on the sea floor since 80 million years ago
Source: Sci Rep. 2016 Apr 14;6:24355. doi: 10.1038/srep24355 (PMC4830954; doi:10.1038/srep24355)
Supplement: Supplementary Information [file srep24355-s1.docx]

**Supplementary Information for:**

**Burrowing hard corals occurring on the sea floor since 80 million years ago**

**Asuka Sentoku**^1, +^**, Yuki Tokuda**^2, 4, *, +^ **and Yoichi Ezaki**^3^

^1^Seto Marine Biological Laboratory, Field Science Education and Research Center, Kyoto University, 459 Shirahama, Nishimuro, Wakayama 649-2211, Japan.

^2^Tottori Prefectural Museum, 2-124 Higashimachi, Tottori 680-0011, Japan.

^3^Department of Geosciences, Faculty of Science, Osaka City University, 3-3-138 Sugimoto, Sumiyoshi-ku, Osaka 558-8585, Japan.

^4^Tottori University of Environmental Studies, 1-1-1 Wakabadaikita, Tottori 689-1111, Japan.

^*^corresponding. tokuda35@gmail.com

^+^these authors contributed equally to this work

**1. Supplementary figures**

**2. Movie Legends**

**1. Supplementary figures**

**
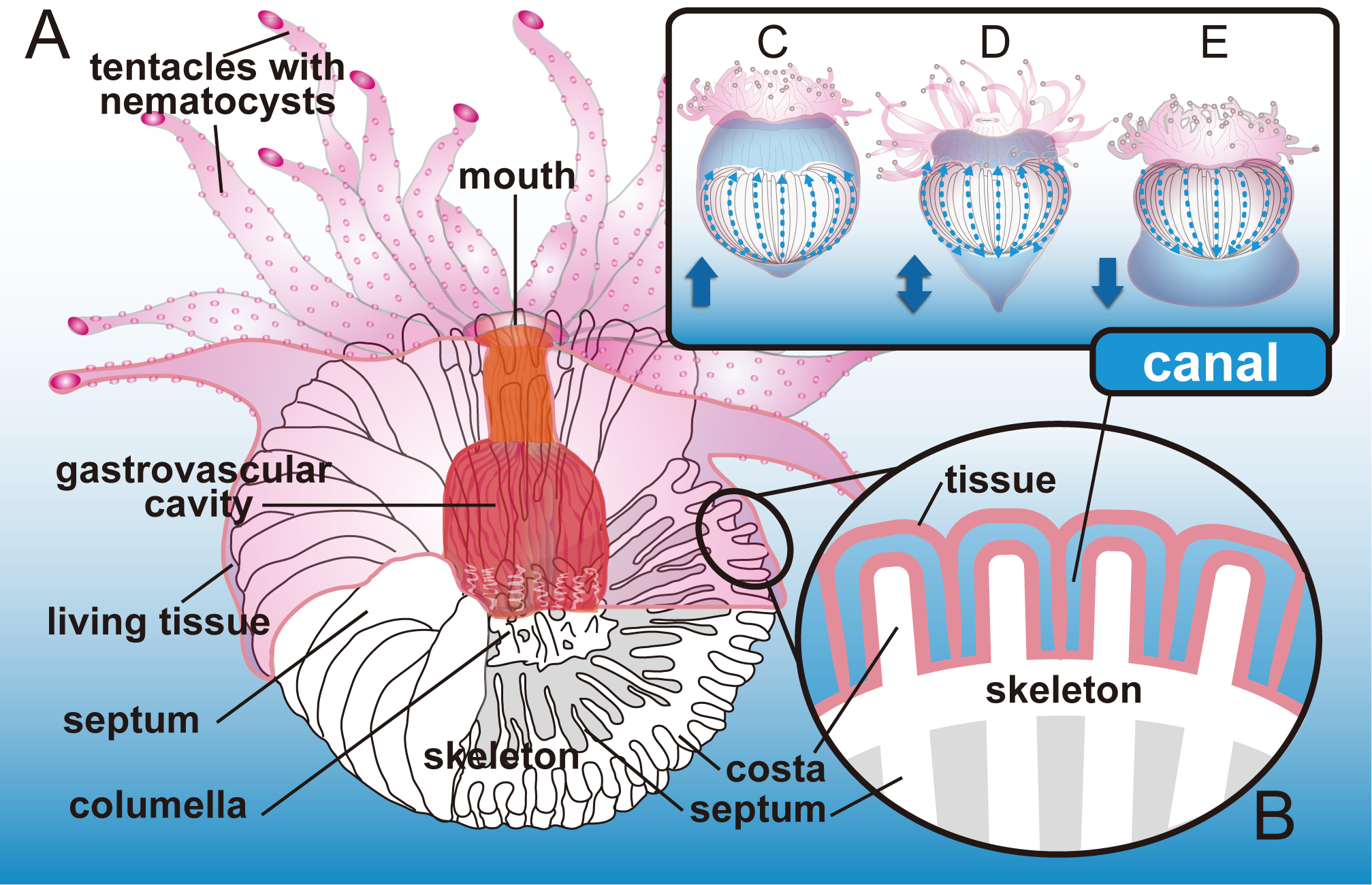
**

**Figure S1.** Schematic diagrams depicting the anatomy and locomotion behaviour of polyps of *Deltocyathoides orientalis*. (A) Diagram depicting both the soft and skeletal parts; (B) Enlarged view of the ellipsis in (A); (C-E) changes in the soft parts of the polyp are dependent on the flow of seawater (indicated by arrows) through the canals.

**
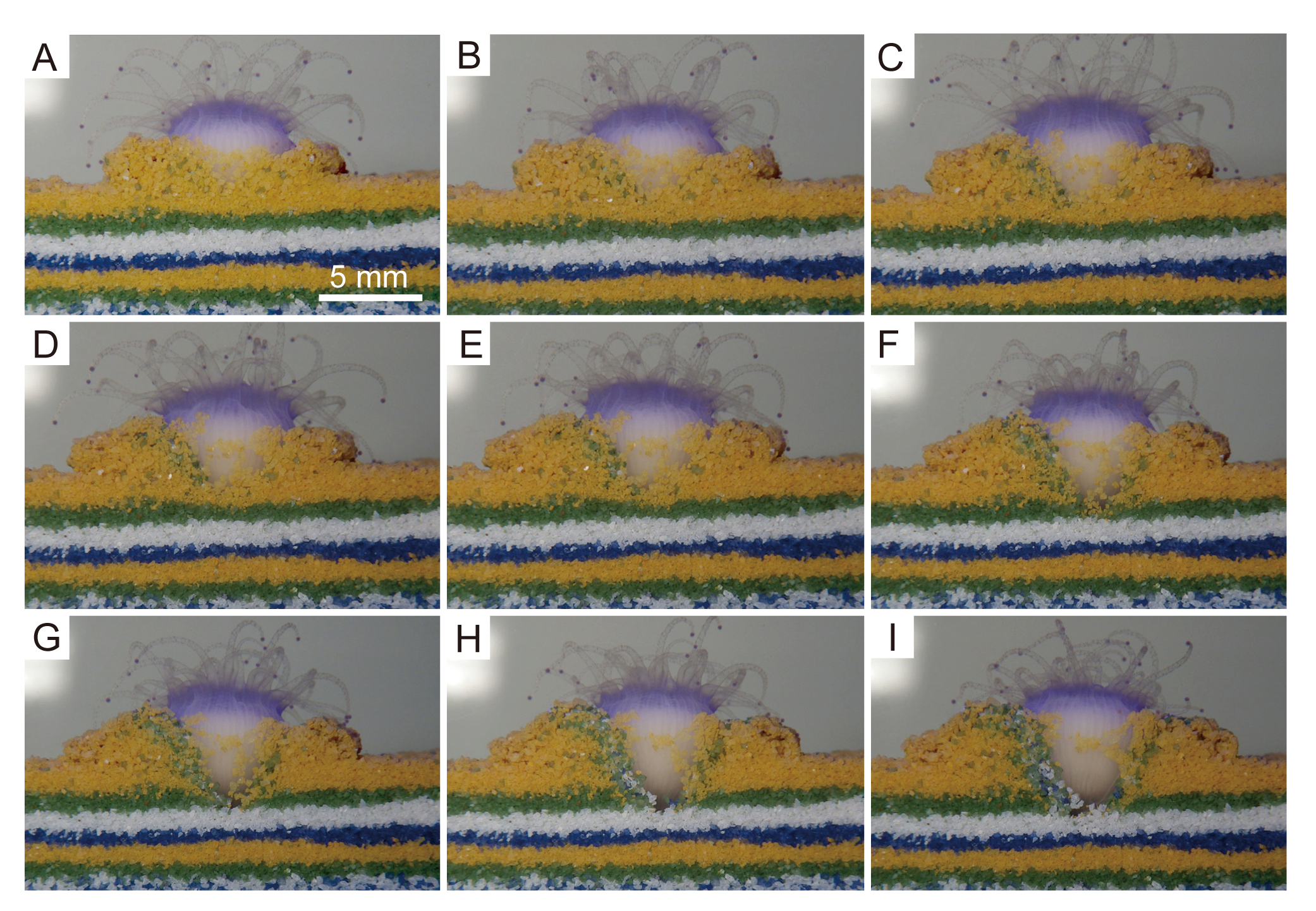
**

**Figure S2.** Time-lapse series of a burrowing polyp. (A) Initial placement of the coral on the substrate (0 min); (B) elapsed time: 200 min; (C) 300 min; (D) 400 min; (E) 500 min; (F) 600 min; (G) 700 min; (H) 800 min; (I) 900 min.

**
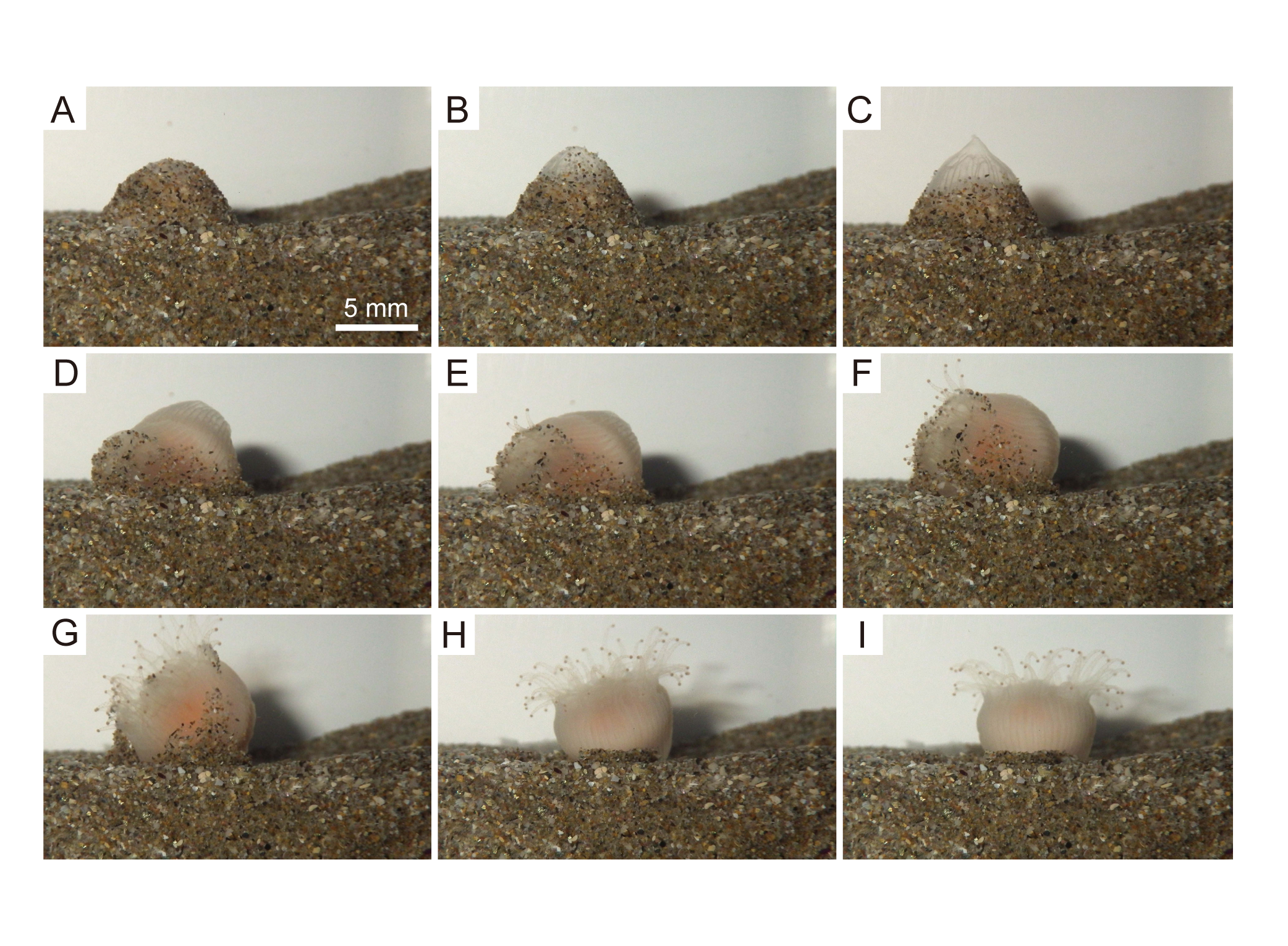
**

**Figure S3.** Time-lapse series demonstrating the righting behaviour of an overturned polyp. (A) Overturned polyp (0 min); (B) elapsed time: 13 min; (C) 43 min; (D) 165 min; (E) 196 min; (F) 210 min; (G) 211 min; (H) 401 min; (I) 426 min.

**
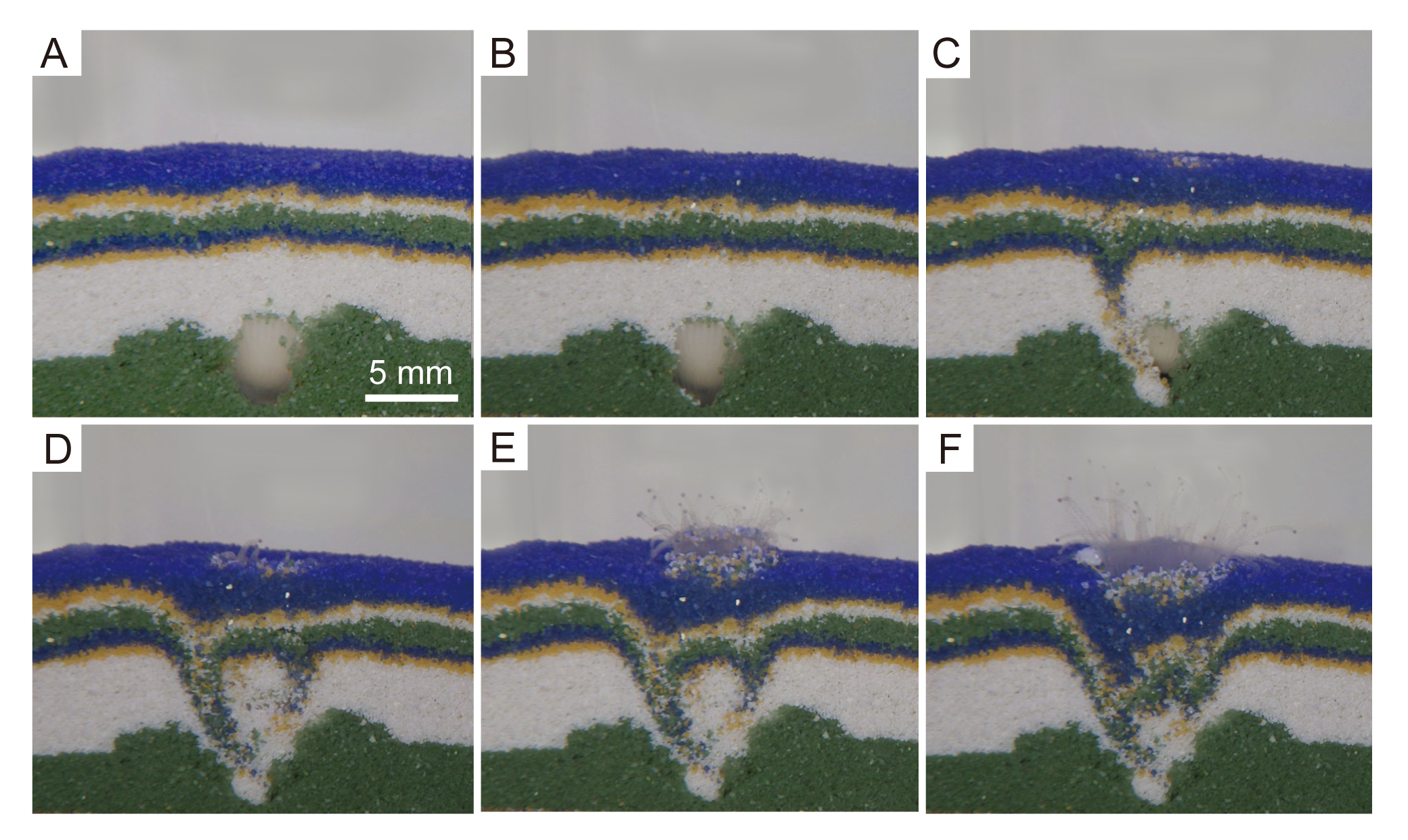
**

**Figure S4.** Time-lapse series demonstrating a coral escaping from burial. (A) Coral buried under layers of differently coloured substrate (0 min); (B) elapsed time: 150 min; (C) 200 min; (D) 250 min; (E) 300 min; (F) 350 min.

**2. Movie Legends**

**Movie S1.** Time-lapse video demonstrating burrowing behaviour of *Deltocyathoides orientalis* on fine sand substrates (984 min in 42 seconds)

**Movie S2.** Time-lapse video demonstrating burrowing behaviour of *Deltocyathoides orientalis* on coloured layer sand (990 min in 66 seconds)

**Movie S3.** Time-lapse video demonstrating righting behaviour of an overturned polyp of *Deltocyathoides orientalis* (495 min in 33 seconds).

**Movie S4.** Time-lapse video demonstrating escaping from burial of *Deltocyathoides orientalis* (990 min in 66 seconds).
